# Xenogeneic Collagen Matrix Versus Free Gingival Graft for Augmenting Peri‐Implant Keratinized Mucosa Around Dental Implants: A Systematic Review and Meta‐Analysis

**DOI:** 10.1002/cre2.932

**Published:** 2024-07-07

**Authors:** Momen A. Atieh, Maanas Shah, Abeer Hakam, Suhailah Alshaali, Reem Kasouha, Andrew Tawse‐Smith, Nabeel H. M. Alsabeeha

**Affiliations:** ^1^ Mohammed Bin Rashid University of Medicine and Health Sciences, Hamdan Bin Mohammed College of Dental Medicine Dubai Healthcare City Dubai UAE; ^2^ Sir John Walsh Research Institute, Faculty of Dentistry University of Otago Dunedin New Zealand; ^3^ School of Dentistry University of Jordan Amman Jordan; ^4^ Department of Dental Services Emirates Health Services Dubai UAE

**Keywords:** dental implant, free gingival graft, keratinized mucosa, meta‐analysis, systematic review, xenogeneic collagen matrix

## Abstract

**Objectives:**

There is a growing evidence to suggest augmenting peri‐implant keratinized mucosa in the presence of ≤ 2 mm of keratinized mucosa. However, the most appropriate surgical technique and augmentation materials have yet to be defined. The aim of this systematic review and meta‐analyses was to evaluate the clinical and patient‐reported outcomes of augmenting keratinized mucosa around implants using free gingival graft (FGG) versus xenogeneic collagen matrix (XCM) before commencing prosthetic implant treatment.

**Material and Methods:**

Electronic databases were searched to identify observational studies comparing implant sites augmented with FGG to those augmented with XCM. The risk of bias was assessed using the Cochrane Collaboration's Risk of Bias tool.

**Results:**

Six studies with 174 participants were included in the present review. Of these, 87 participants had FGG, whereas the remaining participants had XCM. At 6 months, sites augmented with FGG were associated with less changes in the gained width of peri‐implant keratinized mucosa compared to those augmented with XCM (mean difference 1.06; 95% confidence interval −0.01 to 2.13; *p* = 0.05). The difference, however, was marginally significant. The difference between the two groups in changes in thickness of peri‐implant keratinized mucosa at 6 months was statistically significantly in favor of FGG. On the other hand, XCM had significantly shorter surgical time, lower postoperative pain score, and higher color match compared to FGG.

**Conclusions:**

Within the limitation of this review, the augmentation of keratinized mucosa using FGG before the placement of the final prosthesis may have short‐term positive effects on soft tissue thickness. XCM might be considered in aesthetically demanding implant sites and where patient comfort or shorter surgical time is a priority. The evidence support, however, is of low to moderate certainty; therefore, further studies are needed to support the findings of the present review.

## 1 Introduction

The influence of peri‐implant keratinized mucosa on peri‐implant tissue health has been a subject of debate within the implant community for decades. Early reports, mainly on machined surface implants, failed to demonstrate a correlation between the amount of keratinized tissues and peri‐implant soft tissue health or changes in marginal bone levels (Adell et al. 1985, 1986, Bengazi, Wennström, and Lekholm 1996, Lekholm et al. 1996, Wennström, Bengazi, and Lekholm 1994). Hence, a requirement for a minimum width or thickness of keratinized tissues around dental implants has not been established. In recent years, however, a growing body of evidence seems to shift our understanding with findings indicating a strong relationship between the lack of adequate keratinized mucosa and a significant accumulation of biofilm, peri‐implant mucosal inflammation, and recession (Brito et al. 2014, Chung et al. 2006, Grischke et al. 2019, Lin, Chan, and Wang 2013, Perussolo et al. 2018, Schrott et al. 2009). In fact, in the presence of ≤ 2 mm of keratinized mucosa, augmentation procedures were deemed necessary for the maintenance of peri‐implant tissue health (Giannobile et al. 2018, Sanz et al. 2022, Thoma et al. 2021). Moreover, Roccuzzo, Grasso and Dalmasso (2016) recommended the augmentation of keratinized mucosa in cases of ongoing peri‐implant mucosal recession or when patients are experiencing difficulty in maintaining adequate plaque control. Augmentation of keratinized mucosa has also been suggested to improve bleeding and plaque indices post peri‐implant mucositis treatment (Basegmez et al. 2012). In these instances, augmentation with autogenous connective tissue grafts remains the gold standard, with some suggesting xenogeneic‐derived substitutes as alternatives, albeit, with limited evidence.

While there seems to be a general consensus on the need for augmenting keratinized mucosa when insufficient, the appropriate surgical technique or timing of augmentation has yet to be defined. For example, an apically positioned flap has traditionally been used for the augmentation of keratinized mucosa (Friedman 1957). However, the relapse and contraction of the flap (Hillerup 1980) required the addition of autogenous tissue graft to enhance predictability and stability of the augmented mucosa (Basegmez et al. 2012, Hillerup 1980). Such grafts mandated a second surgical site with added postoperative morbidity to patients. Additionally, adequate size of these grafts might not always be available due to anatomical boundaries and their “patch‐like” appearance might not be aesthetically pleasing (Basegmez et al. 2012, Reiser et al. 1996). Therefore, substitutes, such as collagen matrix, have been utilized to overcome these limitations. Collagen matrices are often xenogeneic and consist of two layers, a compact one for wound protection and a porous one for enhancing vascularization and clot stabilization (Ghanaati et al. 2011). Histological studies (Ghanaati et al. 2011, Vignoletti et al. 2011) showed that those matrices can integrate well with the surrounding tissues without any significant inflammatory response. Moreover, soft tissue substitutes were considered safe alternatives that meet the aesthetic expectations of patients and operators (Schmitt et al. 2016). Nevertheless, systematic reviews (Atieh et al. 2016, Huang et al. 2019) on the use of collagen matrices in the treatment of gingival recessions did not demonstrate significant differences between soft tissue substitutes and autogenous tissue grafts in terms of root coverage or gain in keratinized tissue with limited evidence to suggest improved postoperative morbidity or operating time.

Soft tissue augmentation around dental implants can be performed before implant placement, at the time of implant placement, before abutment connection, or after the placement of the final prosthesis. However, a general consensus on the ideal timing for augmenting the peri‐implant keratinized mucosa remains lacking. Nevertheless, there is more emphasis on optimizing the peri‐implant soft tissues before the prosthetic phase of implant treatment to improve future aesthetic outcomes and minimize any biological complications (Lin et al. 2018). Moreover, the outcomes of any augmentation procedure following the insertion of the final prosthesis were shown to be less predictable and were often seen as “rescue” procedures that required advanced surgical skills (Thoma, Muhlemann, and Jung 2014, Thoma et al. 2014). Therefore, the current argument does not revolve around the necessity of a sufficient amount of peri‐implant keratinized mucosa to maintain healthy and stable peri‐implant tissues before completing implant treatment. Instead, the focus is on how we can predictably execute the augmentation of peri‐implant keratinized mucosa before the insertion of the final prosthesis. Numerous clinical studies (Sanz et al. 2009, Schmitt et al. 2016, 2013, Solonko et al. 2022) showed comparable clinical outcomes in terms of gain in width of keratinized mucosa and aesthetic outcomes using different autogenous soft tissue grafts and substitutes. The conclusions, albeit were not decisive, particularly when comparing conventional surgical approaches utilizing a particular autogenous tissue graft or soft tissue substitute and performed at a specific time point during the rehabilitation. Hence, the aim of the present systematic review was to investigate the clinical and patient‐reported outcomes of augmenting keratinized mucosa around implants using free gingival graft (FGG) versus xenogeneic collagen matrix (XCM) before commencing prosthetic implant treatment.

## 2 Materials and Methods

The current systematic review was developed following the guidelines provided by the Cochrane Collaboration (Higgins et al. 2022) and Preferred Reporting Items for Systematic Reviews and Meta‐analyses (Page et al. 2021). The eligibility criteria were defined based on the participant, intervention, comparison, outcome (PICO) framework (Higgins et al. 2022, Richardson et al. 1995):

Participant: Human adult aged ≥ 18 years who required soft tissue augmentation before the placement of final implant prosthesis.

Intervention: Apically positioned flap with XCM.

Comparison: Apically positioned flap with FGG.

Outcomes: Changes in width and thickness of keratinized mucosa, periodontal parameters, aesthetic outcomes, patient‐reported outcome measures and operating time.

The study was registered at the National Institute for Health Research (NHR) under the PROSPERO ID CRD42023456452. Ethical approval and informed consent were not required for this systematic review.

### 2.1 Types of Studies

#### 2.1.1 Inclusion Criteria

This review included randomized and non‐randomized clinical studies, with at least 10 participants and 1 month of follow‐up, comparing the use of XCM with FGG for augmenting keratinized mucosa around dental implants before the placement of final prosthesis. The included studies must report on changes in width and/or thickness of keratinized mucosa, periodontal parameters, aesthetic outcomes, patient‐reported outcome measures, or operating time. No language restrictions or publication status were employed. Non‐randomized studies were included to supplement the evidence and address long‐term outcomes (Higgins et al. 2022).

### 2.2 Exclusion Criteria

Studies that evaluated the use of XCM before implant placement, at implant placement, after placement of permanent prosthesis or during maintenance were excluded. Studies that did not include a control group were also excluded.

### 2.3 Type of Participants

Participants who were 18 years of age or older and had dental implants with shallow vestibule, insufficient keratinized mucosa and required soft tissue augmentation at the time of uncovering implant(s) or before abutment connection.

### 2.4 Types of Interventions

The intervention group involved the use of either porcine‐ or bovine‐derived XCM with apically positioned flap while the control group involved the use of FGG with apically positioned flap.

### 2.5 Outcome Measures

#### 2.5.1 Primary Outcome

Changes in of keratinized mucosa are the primary outcome.

### 2.6 Secondary Outcomes

The secondary outcomes are as follows:

Changes in thickness of keratinized mucosa.

Changes in periodontal parameters (probing pocket depths, modified bleeding, and plaque indices).

Aesthetic outcomes (changes in color, texture, and contour).

Patient‐reported outcome measures.

Operating time.

### 2.7 Search Strategy

The search protocol followed standard procedures (Faggion, Atieh, and Park 2013, Higgins et al. 2022). The following electronic databases were searched for ongoing and unpublished trials up to August 10, 2023: MEDLINE, EMBASE, The Cochrane Central Register of Controlled Trials (CENTRAL), MetaRegister, ClinicalTrials.gov, and the System for Information on Grey Literature in Europe (http://www.opengrey.eu) (Table 1). The search was performed independently and in duplicate by two authors (M.A.A. and N.H.M.A.). Manual search of the last 5 years of relevant dental journals (*Clinical Implant Dentistry and Related Research*, *Clinical Oral Implants Research*, *Implant Dentistry*, *International Journal of Oral and Maxillofacial Implants*, *International Journal of Periodontics and Restorative Dentistry*, *Journal of Clinical Periodontology*, and *Journal of Periodontology*) and bibliographies of all eligible papers were also carried out for additional studies.

**Table 1:** Databases and search terms.

Databases and search terms	Keywords
Published studies	
PubMed (August 10, 2023)	(collagen matrix OR free gingival graft OR keratinized mucosa OR keratinized tissue) AND (dental implant* OR oral implant*) AND (tissue augmentation OR tissue augmentation)
EMBASE via Ovid (August 10, 2023)	(collagen adj matrix).mp. OR (free adj gingival adj graft).mp. OR (keratinized adj mucosa).mp. OR(keratinized adj tissue).mp. AND (dental adj implant).mp. OR (oral adj implant).mp. AND (tissue adj augmentation).mp. OR (tissue adj augmentation).mp
Cochrane Central Register of Controlled Trials (CENTRAL) via Ovid (August 10, 2023)	(collagen adj matrix).mp. OR (free adj gingival adj graft).mp. OR (keratinized adj mucosa).mp. OR(keratinized adj tissue).mp. AND (dental adj implant).mp. OR (oral adj implant).mp. AND (tissue adj augmentation).mp. OR (tissue adj augmentation).mp
Unpublished studies	
ClinicalTrials.gov (August 10, 2023)	(collagen adj matrix).mp. OR (free adj gingival adj graft).mp. OR (keratinized adj mucosa).mp. OR(keratinized adj tissue).mp. AND (dental adj implant).mp. OR (oral adj implant).mp. AND (tissue adj augmentation).mp. OR (tissue adj augmentation).mp

### 2.8 Selection of Studies

Two reviewers (M.A.A. and N.H.M.A.) independently and in duplicate examined the retrieved citations on the basis of the title, abstract, and keywords. Irrelevant papers were excluded, and the full texts of the remaining ones were obtained. An eligibility form was used to examine papers for inclusion in the review. Any disagreements were resolved by discussion to reach a consensus or by consultation with a third reviewer (M.S.). In the event of duplicate papers, the one with the most relevant and sufficient information was selected. All the reasons for exclusion were reported.

### 2.9 Data Collection

Two authors (M.A.A. and N.H.M.A.) used a data extraction form and independently collected the following information from the included studies: (1) Study characteristics: Title, authors' names, study location, language of publication, year of publication, published or unpublished data, source of study funding, and study design. (2) Participants: Demographic characteristics, inclusion/exclusion criteria, number of participants in test and control groups, attrition rate, and reasons for dropouts. (3) Interventions: Number of participants undergoing soft tissue augmentation with XCM. (4) Comparison: Number of participants undergoing soft tissue augmentation with FGG. (5) Outcomes: Changes in width and thickness of keratinized mucosa, changes in periodontal parameters, aesthetic outcomes, patient‐reported outcome measures, and operating time. (6) Length of the observation period. Any disagreements between reviewers were resolved by discussion to reach a consensus or by consultation with a third reviewer (M.S.). Corresponding authors were contacted for additional information if required.

### 2.10 Quality Assessment of Included Studies

Two reviewers (M.A.A. and N.H.M.A.) used the Cochrane Collaboration's Risk of Bias tool for randomized trials (RoB 2) and the Risk Of Bias In Non‐randomized Studies of Interventions (ROBINS‐I) tool (Higgins et al. 2022, Sterne et al. 2016) to assess all the included studies independently and in duplicate.

### 2.11 Data Synthesis

A statistical software program (Review Manager [RevMan] software, version 5.3, The Nordic Cochrane Centre, The Cochrane Collaboration, Copenhagen, Denmark) was used to conduct meta‐analyses for studies of similar comparisons reporting the same outcome measures. For example, continuous data, such as changes in width of keratinized mucosa, were expressed in mean difference (MD) or standardized mean difference (SMD) and 95% confidence intervals (CIs). Random‐effects model was used to pool the results from more than one study as heterogeneity between studies was expected. With fewer than 10 studies, publication bias was not formally assessed because the power to detect publication bias was limited (Higgins et al. 2022). The statistical heterogeneity across different studies was assessed by means of Cochran's test for heterogeneity and *I* ^2^ statistic (Higgins et al. 2022). An *I* ^2^ value of > 50 indicated a substantial heterogeneity. The participant was considered as the statistical unit of analysis. To deal with within‐patient correlation of clinical outcomes, we set the within‐patient correlation coefficient of 0.9 and inflated the standard error where studies did not account for within‐patient correlation (Higgins et al. 2022). In dealing with within‐patient correlation, we followed the methods for dealing with clustered trials suggested in chapter 23 of the Cochrane Handbook (Higgins et al. 2022).

A leave‐one study‐out sensitivity analysis was conducted to check the source of heterogeneity, stability of results and influence of studies. Sensitivity analysis was conducted to assess whether estimated effects differ when we exclude studies at high risk of bias from analyses. The certainty of evidence was assessed using the five GRADE criteria (risk of bias, inconsistency, imprecision, indirectness, and publication bias) (Higgins et al. 2022). A software program (GRADEpro Guideline Development Tool software, McMaster University and Evidence Prime, 2021, available from https://gradepro.com) was used to create the summary of findings table.

## 3 Results

### 3.1 Characteristics of the Study Settings

**Figure 1:**
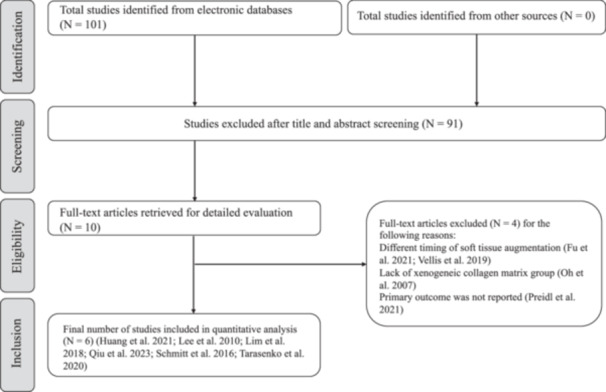
Flowchart of the search process.

A total of 101 studies were retrieved from the databases (Figure 1). After titles and abstracts were examined independently and in duplicate by two review authors (M.A.A. and N.H.M.A.), 10 studies were eligible for full‐text review (Fu et al. 2021, Huang et al. 2021, Lee, Kim, and Jang 2010, Lim, An, and Lee 2018, Oh et al. 2017, Preidl et al. 2021, Qiu et al. 2023, Schmitt et al. 2016, Vellis, Kutkut, and Al‐Sabbagh 2019, Tarasenko et al. 2020). Four studies (Fu et al. 2021, Oh et al. 2017, Preidl et al. 2021, Vellis, Kutkut, and Al‐Sabbagh 2019) were subsequently excluded and as a result six studies (Huang et al. 2021, Lee, Kim, and Jang 2010, Lim, An, and Lee 2018, Qiu et al. 2023, Schmitt et al. 2016, Tarasenko et al. 2020) were included in the present review (Table 2). The main reason for exclusion was the timing of soft tissue augmentation, which was performed either before implant placement or during the maintenance phase. Of the six included studies, two were conducted in China (Huang et al. 2021, Qiu et al. 2023), two in Korea (Lee, Kim, and Jang 2010, Lim, An, and Lee 2018), one in Germany (Schmitt et al. 2016), and one in Russia (Tarasenko et al. 2020). All the included studies, except one (Lim, An, and Lee 2018), were conducted in a university setting and all were parallel‐group studies. None of the included studies received funding or support from the industry or material manufacturers.

**Table 2:** Characteristics of the included studies.

	Huang et al. (2021)	Lee, Kim, and Jang (2010)	Lim, An, and Lee (2018)	Qiu et al. (2023)	Schmitt et al. (2016)	Tarasenko et al. (2020)
Study design	RCT	CS	RS	RCT	CCT	RCT
Study location	Department of Periodontology, Stomatology Hospital, School of Stomatology, Zhejiang University School of Medicine, Hangzhou, China	Department of Periodontology, Chosun University School of Dentistry, Gwangju, Korea	Department of Periodontology, Veterans Health Service Medical Center, Seoul, Korea	Department of Oral Implantology, Tianjin Stomatological Hospital, Nankai University, Tianjin, China	Department of Oral and Maxillofacial Surgery, University of Erlangen‐Nuremberg, Erlangen, Germany	Department of Dental Surgery, Sechenov University, Moscow, Russia
Number of participants/implants evaluated *N*						
XCM	12/19	3/3^[Link]^, ^a^	11/30^[Link]^, ^a^	15/26	27/102	19/53^[Link]^, ^a^
FGG	13/19	3/8	14/31	15/26	21/74	21/49
Age (years), mean ± SD						
XCM	53.00 ± 15.00	NR	60.90 ± 8.90	35.20 ± 6.84	NR	51.50 ± 10.90
FGG	49.00 ± 13.00	NR	63.20 ± 9.90	36.80 ± 5.89	NR	52.20 ± 8.70
Implant location	Mandible and maxilla	Posterior mandible and maxilla	Posterior mandible	Posterior mandible	Anterior mandible	Posterior mandible
Donor site (source of graft)						
XCM	Porcine CM^b^	Bovine CM^c^	Porcine CM^b^	Porcine CM^b^	Porcine CM^b^	Porcine CM^b^
FGG	Palate	Palate	Palate	Palate	Palate	Palate
Methods of assessment	Manual periodontal probe, UNC 15^d^ Endodontic file with a rubber stop VAS Aesthetic score	Manual periodontal probe, PCP 10^d^	Manual periodontal probe, PCP 12^d^ Standardized photos	Manual periodontal probe, UNC 15^d^ CBCT Standardized photos VAS	Manual periodontal probe, PCP 12^d^	Caliper Manual periodontal probe Manual periodontal probe with rubber stop VAS
Changes in width of peri‐implant keratinized mucosa (mm) at 1 month						
XCM	NR	1.83 ± 1.04	NR	4.18 ± 1.04	9.84 ± 2.85	3.03 ± 0.58
FGG	NR	2.50 ± 1.00	NR	4.23 ± 0.77	10.23 ± 2.46	4.85 ± 1.11
Changes in width of peri‐implant keratinized mucosa (mm) at 2–3 months						
XCM	1.60 ± 0.90	NR	NR	3.54 ± 0.94	8.69 ± 2.72	NR
FGG	4.10 ± 1.40	NR	NR	3.86 ± 0.82	9.39 ± 2.66	NR
Changes in width of peri‐implant keratinized mucosa (mm) at 6 months						
XCM	1.80 ± 1.00	NR	4.70 ± 1.05	3.28 ± 0.96	7.75 ± 2.75	2.51 ± 0.60
FGG	4.10 ± 1.60	NR	4.37 ± 0.81	3.60 ± 0.79	8.83 ± 2.71	4.47 ± 1.10
Changes in width of peri‐implant keratinized mucosa (mm) at 12 months						
XCM	NR	NR	3.73 ± 0.97	NR	7.13 ± 2.63	NR
FGG	NR	NR	4.10 ± 1.16	NR	8.46 ± 2.68	NR
Changes in thickness of peri‐implant keratinized mucosa (mm) at 6 months						
XCM	0.10 ± 0.50	NR	NR	0.95 ± 0.29	NR	NR
FGG	0.90 ± 0.50	NR	NR	1.24 ± 0.34	NR	NR
Changes in probing pocket depth (mm) at 2–3 months						
XCM	1.42 ± 0.46	NR	NR	2.08 ± 0.33	NR	NR
FGG	1.41 ± 0.39	NR	NR	2.26 ± 0.34	NR	NR
Changes in probing pocket depth (mm) at 6 months						
XCM	1.45 ± 0.54	NR	NR	1.98 ± 0.35	NR	NR
FGG	1.36 ± 0.35	NR	NR	2.13 ± 0.23	NR	NR
Changes in modified sulcus bleeding index at 1–3 months						
XCM	0.26 ± 0.37	NR	NR	0.01 ± 0.06	NR	NR
FGG	0.14 ± 0.28	NR	NR	0.01 ± 0.06	NR	NR
Changes in modified sulcus bleeding index at 6 months						
XCM	0.33 ± 0.64	NR	NR	0.00 ± 0.00	NR	NR
FGG	0.11 ± 0.27	NR	NR	0.00 ± 0.00	NR	NR
Changes in modified plaque index at 3 months						
XCM	NR	NR	NR	0.19 ± 0.22	NR	NR
FGG	NR	NR	NR	0.19 ± 0.24	NR	NR
Changes in modified plaque index at 6 months						
XCM	NR	NR	NR	0.10 ± 0.15	NR	NR
FGG	NR	NR	NR	0.11 ± 0.20	NR	NR
Changes in color						
XCM	1.90 ± 0.30	NR	3.30 ± 0.50	3.78 ± 0.44	NR	NR
FGG	1.40 ± 0.60	NR	2.50 ± 0.90	1.56 ± 0.53	NR	NR
Changes in texture						
XCM	1.60 ± 0.30	NR	2.80 ± 0.80	3.67 ± 0.50	NR	NR
FGG	1.90 ± 0.30	NR	1.80 ± 0.40	2.44 ± 0.53	NR	NR
Changes in contour						
XCM	1.20 ± 0.60	NR	2.70 ± 0.50	NR	NR	NR
FGG	1.40 ± 0.40	NR	2.10 ± 0.60	NR	NR	NR
PROMs						
Pain						
XCM	2.60 ± 2.30	NR	NR	2.89 ± 1.69	NR	1.06 ± 1.43
FGG	3.40 ± 1.80	NR	NR	4.56 ± 1.33	NR	4.84 ± 2.00
Satisfaction						
XCM	9.70 ± 0.60	NR	NR	6.53 ± 0.71	NR	NR
FGG	9.60 ± 0.60	NR	NR	4.13 ± 1.16	NR	NR
Operating time (min)						
XCM	39.00 ± 8.00	NR	NR	NR	65.11 ± 15.36	NR
FGG	60.00 ± 9.00	NR	NR	NR	84.33 ± 14.23	NR
Follow‐up period (months)	6	1	12	6	60	6

Abbreviations: CBCT: cone beam computed tomography; CCT: controlled clinical trial; CS: case series; FGG: free gingival graft; NR: not reported; PROMs: patient‐reported outcome measures; RCT: randomized controlled trial; RS: retrospective study; VAS: visual analogue scale; XCM: xenogeneic collagen matrix.

^a^ Only data that are related to XCM and FGG groups were included.

^b^ Mucograft, Geistlich Pharma AG, Wolhusen, Switzerland.

^c^ Collatape, Zimmer Dental, Carlsbad, CA, USA.

^d^ Hu‐Friedy Manufacturing Co., Chicago, IL, USA.

### 3.2 Characteristics of Participants at Baseline

The inclusion criteria were as follows:

1. Aged ≥ 18 (Huang et al. 2021, Schmitt et al. 2016, Tarasenko et al. 2020).
2. Systemically healthy participants (Huang et al. 2021, Lee, Kim, and Jang 2010, Lim, An, and Lee 2018, Tarasenko et al. 2020).
3. At least one implant site that had shallow vestibule and ≤ 2 mm of keratinized tissue and required soft tissue augmentation before commencing restorative treatment (Huang et al. 2021, Lee, Kim, and Jang 2010, Lim, An, and Lee 2018, Qiu et al. 2023, Schmitt et al. 2016, Tarasenko et al. 2020).
4. Adequate plaque control (Schmitt et al. 2016) or having a full‐mouth plaque score ≤ 20% (Qiu et al. 2023, Tarasenko et al. 2020).

The exclusion criteria were as follows:

1. Systemic conditions and/or medications that may interfere with healing (Huang et al. 2021, Lim, An, and Lee 2018, Qiu et al. 2023, Schmitt et al. 2016).
2. Immunocompromised status (Huang et al. 2021, Tarasenko et al. 2020).
3. History of radiotherapy (Huang et al. 2021).
4. Allergy to collagen (Huang et al. 2021).
5. Smoking (Huang et al. 2021, Schmitt et al. 2016) or smoking > 10 cigarettes/day (Qiu et al. 2023, Tarasenko et al. 2020).
6. Pregnant or lactating women (Huang et al. 2021, Qiu et al. 2023, Tarasenko et al. 2020).
7. Untreated periodontal diseases (Huang et al. 2021, Schmitt et al. 2016).
8. History of mucogingival surgery (Huang et al. 2021).

### 3.3 Characteristics of the Interventions

All participants had implants that were planned for second‐stage implant surgery. The augmentation of the keratinized mucosa was carried out either before (Qiu et al. 2023, Tarasenko et al. 2020) or at the time of uncovering the implants (Lee, Kim, and Jang 2010, Lim, An, and Lee 2018, Schmitt et al. 2016). The surgical procedure was relatively similar across the included studies. Before surgery, prophylactic antibiotics were administered (Qiu et al. 2023), and participants were asked to rinse with 0.12% chlorhexidine mouthwash for 30 s (Qiu et al. 2023) or 60 s (Lim, An, and Lee 2018, Tarasenko et al. 2020). The flap design consisted of mid‐crestal horizontal incision with two bilateral vertical incisions extending beyond the mucogingival junction at the implant site (Huang et al. 2021, Lee, Kim, and Jang 2010, Lim, An, and Lee 2018, Qiu et al. 2023, Schmitt et al. 2016, Tarasenko et al. 2020). A partial thickness flap was raised and carefully dissected, then moved apically along the mucogingival junction, and fixed to the periosteum with interrupted sutures to create a new vestibule (Huang et al. 2021, Lee, Kim, and Jang 2010, Lim, An, and Lee 2018, Qiu et al. 2023, Schmitt et al. 2016, Tarasenko et al. 2020). In randomized controlled trials (Huang et al. 2021, Qiu et al. 2023, Tarasenko et al. 2020), the allocation to a specific treatment group was conducted following the preparation of the recipient bed. In the FGG group, the graft was harvested from the palate (Huang et al. 2021, Lee, Kim, and Jang 2010, Lim, An, and Lee 2018, Qiu et al. 2023, Schmitt et al. 2016, Tarasenko et al. 2020), thinned to appropriate thickness, and then stabilized on the periosteal bed using crossed mattress and interrupted sutures (Huang et al. 2021, Lim, An, and Lee 2018, Qiu et al. 2023, Schmitt et al. 2016). An absorbable gelatin sponge (Huang et al. 2021, Qiu et al. 2023), hemostatic collagen membrane (Tarasenko et al. 2020), or a bandage splint (Schmitt et al. 2016) was used to cover the donor site. As for the XCM group, the XCM was trimmed to the appropriate size and then stabilized to the recipient bed in a similar fashion (Lee, Kim, and Jang 2010, Lim, An, and Lee 2018, Qiu et al. 2023, Schmitt et al. 2016). Postoperatively, antibiotics (Huang et al. 2021, Lee, Kim, and Jang 2010, Lim, An, and Lee 2018, Qiu et al. 2023), analgesics (Huang et al. 2021, Lim, An, and Lee 2018), nonsteroidal anti‐inflammatory drugs (Schmitt et al. 2016, Tarasenko et al. 2020), antiseptic mouthwash (Huang et al. 2021, Lee, Kim, and Jang 2010, Lim, An, and Lee 2018, Schmitt et al. 2016, Tarasenko et al. 2020), and revised oral home care (Huang et al. 2021, Lim, An, and Lee 2018, Qiu et al. 2023, Schmitt et al. 2016) were provided to participants of both groups. In 10–14 days, the sutures were removed (Huang et al. 2021, Lee, Kim, and Jang 2010, Lim, An, and Lee 2018, Qiu et al. 2023, Schmitt et al. 2016), and implant restoration was delivered after two (Lim, An, and Lee 2018) to 3 months (Qiu et al. 2023).

### 3.4 Characteristics of Outcome Measures

The primary outcome measures were as follows:

- Changes in width of peri‐implant keratinized mucosa, as measured by a periodontal probe (Huang et al. 2021, Lee, Kim, and Jang 2010, Lim, An, and Lee 2018, Qiu et al. 2023, Schmitt et al. 2016, Tarasenko et al. 2020).

The secondary outcome measures were as follows:

- Changes in thickness of peri‐implant keratinized mucosa at 6 months, as measured by endodontic file (Huang et al. 2021) or cone beam computed tomography (Qiu et al. 2023).
- Changes in probing pocket depths, as measured by a periodontal probe (Huang et al. 2021, Qiu et al. 2023).
- Changes in modified sulcus bleeding index, as measured by a periodontal probe (Huang et al. 2021, Qiu et al. 2023).
- Changes in modified plaque index, as measured by a periodontal probe (Qiu et al. 2023).
- Aesthetic outcomes (changes in color, texture, and contour), as assessed by using standardized clinical photos and scoring tools (Huang et al. 2021, Lim, An, and Lee 2018, Qiu et al. 2023).
- Pain and satisfaction scores, as measured by visual analogue scale (Huang et al. 2021, Qiu et al. 2023, Tarasenko et al. 2020).
- Operating time, as recorded by a digital timer (Huang et al. 2021, Schmitt et al. 2016).

### 3.5 Risk of Bias in Randomized Controlled Trials

Three studies (Huang et al. 2021, Qiu et al. 2023, Tarasenko et al. 2020) were randomized controlled trials that were judged to be at low risk. They have adequately described the methods of randomization and allocation concealment, reported on masking the data assessors and showed no deviations from intended interventions (Table 3).

**Table 3:** Assessment of risk of bias of the included randomized controlled trials.

	Huang et al. (2021)	Qiu et al. (2023)	Tarasenko et al. (2020)
Bias arising from the randomization process	Low risk	Low risk	Low risk
	Randomization method:	Randomization method:	Randomization method:
	Reported in the article, “The included patients were randomly divided into two groups in 1:1 ratio using a computer‐generated randomization schedule by the investigators”	Reported in the article, “The enrolled patients were randomly divided into two groups in 1:1 ratio using digital software allocation”	Reported in the article, “Randomization of the subjects was done using computer‐generated tables”
	Allocation concealment:	Allocation concealment:	Allocation concealment:
	Reported in the article, “Allocation concealment was performed by sequentially coded, sealed, opaque envelopes, which contained the treatment assignment”	Reported in the article, “Allocation to the treatment groups was concealed from the surgeon by using sealed envelopes”	Reported in the article, “At the time of surgery, one operator opened an envelope containing information about the allocation group and communicated it to the surgeon”
Bias due to deviations from intended interventions	Low risk	Low risk	Low risk
	No deviations arose because of trial context	No deviations arose because of trial context	No deviations arose because of trial context
Bias due to missing outcome data	Low risk	Low risk	Low risk
	All data presented	All data presented	All data presented
Bias in measurement of the outcomes	Low risk	Low risk	Low risk
	Reported in the article, “The clinical outcomes were measured by the same investigator who was masked to the intervention assignment”	Reported in the article, “The group allocation was also concealed for the evaluating examiner and statistician”	Reported in the article “One identification number was assigned to each case to allow blinding in data collection and data analysis”
Bias in selection of the reported results	Low risk	Low risk	Low risk
	All outcomes appear to be detected	All outcomes appear to be detected	All outcomes appear to be detected
Overall risk of bias	Low risk	Low risk	Low risk

### 3.6 Risk of Bias in Non‐Randomized Studies

**Figure 2:**
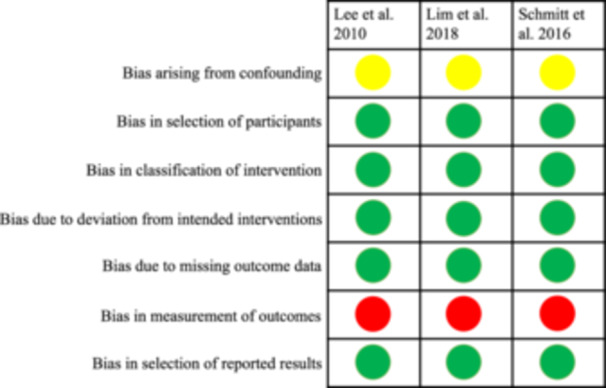
Assessment of risk of bias of the included non‐randomized studies presented with low (green), moderate (yellow), and high (red) risk of bias.

Overall, the remaining three studies (Lee, Kim, and Jang 2010, Lim, An, and Lee 2018, Schmitt et al. 2016) were judged to be at high risk (Figure 2).

#### 3.6.1 Bias due to Confounding

None of the studies showed any attempt to control for confounding. Nevertheless, none of the participants switched between interventions and baseline treatment was not influenced by prognostic variables. Therefore, all studies (Lee, Kim, and Jang 2010, Lim, An, and Lee 2018, Schmitt et al. 2016) were judged to be at moderate risk of bias.

#### 3.6.2 Bias in Selection of Participants Into the Study

None of the studies excluded eligible participants but rather included all participants having implant treatment with insufficient keratinized mucosa before prosthetic rehabilitation. Therefore, all studies (Lee, Kim, and Jang 2010, Lim, An, and Lee 2018, Schmitt et al. 2016) were judged to be at low risk of bias for this domain.

#### 3.6.3 Bias in Classification of Intervention

All the studies (Lee, Kim, and Jang 2010, Lim, An, and Lee 2018, Schmitt et al. 2016) clearly defined the intervention and hence were judged to be at low risk of bias for this domain.

#### 3.6.4 Bias in Measurement of the Outcomes

The three studies (Lee, Kim, and Jang 2010, Lim, An, and Lee 2018, Schmitt et al. 2016) were rated at high risk of bias as none of those studies reported on masking the data assessors.

#### 3.6.5 Bias due to Deviation From Intended Interventions, Incomplete or Missing Outcome Data, or Selection of the Reported Results

None of the three studies (Lee, Kim, and Jang 2010, Lim, An, and Lee 2018, Schmitt et al. 2016) deviated from intended intervention, showed high risk of attrition, or bias due to selective reporting. Thus, they were rated at low risk.

### 3.7 Sample Size Calculation

The three randomized controlled trials (Huang et al. 2021, Qiu et al. 2023, Tarasenko et al. 2020) reported on the sample size calculation.

### 3.8 Clinical Trial Registration

Two studies (Huang et al. 2021, Qiu et al. 2023) were registered in the Chinese clinical trial registry before the initiation of the study.

### 3.9 Effects of Interventions

In total, 174 participants were included in the present review. Of these, 87 participants had XCM while the remaining participants had FGG (Table 4). All the studies reported the data at the participant level.

**Table 4:** Summary of findings.

Outcome	Number of participants (studies)	Relative effect (95% CI)	Anticipated absolute effects^a^ (95% CI)		Certainty of the evidence (GRADE)^b^
			XCM	FGG	
Changes in width of peri‐implant keratinized mucosa at 1 month (mm)	124 (4 studies)	Not estimable	The mean change ranged across this group from 1.83 to 9.84	MD 0.83 higher (0.34 lower to 1.99 higher)	⊕⊕⊕⊝ MODERATE^c^
Changes in width of peri‐implant keratinized mucosa at 2–3 months	103 (3 studies)	Not estimable	The mean change ranged across this group from 1.60 to 8.69	MD 1.23 higher (0.46 lower to 2.92 higher)	⊕⊕⊝⊝ LOW^c^, ^d^
Changes in width of peri‐implant keratinized mucosa at 6 months	168 (5 studies)	Not estimable	The mean change ranged across this group from 1.80 to 7.75	MD 1.06 higher (0.01 lower to 2.13 higher)	⊕⊕⊝⊝ LOW^c^, ^d^
Changes in width of peri‐implant keratinized mucosa at 12 months	74 (2 studies)	Not estimable	The mean change ranged across this group from 3.73 to 7.13	MD 0.53 higher (0.62 lower to 1.68 higher)	⊕⊕⊝⊝ LOW^c^, ^e^
Changes in thickness of peri‐implant keratinized mucosa at 6 months	55 (2 studies)	Not estimable	The mean change ranged across this group from 0.1 to 0.95	MD 0.51 higher (0.01 higher to 1.00 higher)	⊕⊕⊝⊝ LOW^d^, ^e^
Changes in probing pocket depths at 2–3 months	55 (2 studies)	Not estimable	The mean change ranged across this group from 1.42 to 2.08	MD 0.11 lower (0.36 lower to 0.13 higher)	⊕⊕⊕⊝ MODERATE^e^
Changes in probing pocket depths at 6 months	55 (2 studies)	Not estimable	The mean change ranged across this group from 1.45 to 1.98	MD 0.08 lower (0.31 lower to 0.15 higher)	⊕⊕⊕⊝ MODERATE^e^
Changes in modified sulcus bleeding index at 1–3 months	55 (2 studies)	Not estimable	The mean change ranged across this group from 0.01 to 0.26	MD 0.00 (0.05 lower to 0.06 higher)	⊕⊕⊕⊝ MODERATE^e^
Changes in modified sulcus bleeding index at 6 months	55 (2 studies)	Not estimable	The mean change ranged across this group from 0.00 to 0.33	MD 0.12 higher (0.35 lower to 0.59 higher)	⊕⊕⊕⊝ MODERATE^e^
Changes in color	80 (3 studies)	Not estimable	The mean change ranged across this group from 1.90 to 3.78	MD 1.19 lower (2.40 lower to 0.03 higher)	⊕⊕⊝⊝ LOW^c^, ^d^
Changes in texture	80 (3 studies)	Not estimable	The mean change ranged across this group from 1.60 to 3.67	MD 0.62 lower (1.76 lower to 0.52 higher)	⊕⊕⊝⊝ LOW^c^, ^d^
Changes in contour	50 (2 studies)	Not estimable	The mean change ranged across this group from 1.20 to 2.70	MD 0.19 lower (0.97 lower to 0.60 higher)	⊕⊕⊝⊝ LOW^c^, ^d^
Postoperative pain	95 (3 studies)	Not estimable	The mean change ranged across this group from 1.06 to 2.89	MD 2.16 higher (0.45 higher to 3.85 higher)	⊕⊕⊕⊝ MODERATE^e^
Postoperative satisfaction	55 (2 studies)	Not estimable	The mean change ranged across this group from 6.53 to 9.70	MD 1.22 lower (3.48 lower to 1.03 higher)	⊕⊕⊝⊝ LOW^d^, ^e^
Operating time	73 (2 studies)	Not estimable	The mean change ranged across this group from 39.00 to 65.11	MD 20.62 higher (13.46 higher to 27.78 higher)	⊕⊕⊝⊝ LOW^c^, ^e^

*Note:* GRADE Working Group grades of evidence: High certainty: We are very confident that the true effect lies close to that of the estimate of the effect. Moderate certainty: We are moderately confident in the effect estimate: The true effect is likely to be close to the estimate of the effect, but there is a possibility that it is substantially different. Low certainty: Our confidence in the effect estimate is limited: The true effect may be substantially different from the estimate of the effect. Very low certainty: We have very little confidence in the effect estimate: The true effect is likely to be substantially different from the estimate of effect.

Abbreviations: CI: confidence interval; FGG: free gingival graft; MD: mean difference; XCM: xenogeneic collagen matrix.

^a^ The risk in the intervention group (and its 95% CI) is based on the assumed risk in the comparison group and the relative effect of the intervention (and its 95% CI).

^b^ None of the studies suffered from indirectness or detected publication bias.

^c^ Downgraded one level due to risk of bias: At least one study had no blinding.

^d^ Downgraded one level due to inconsistency: Substantial heterogeneity was detected.

^e^ Downgraded one level due to imprecision: The effect estimate is based on two studies.

#### 3.9.1 Changes in Width of Peri‐Implant Keratinized Mucosa

**Figure 3:**
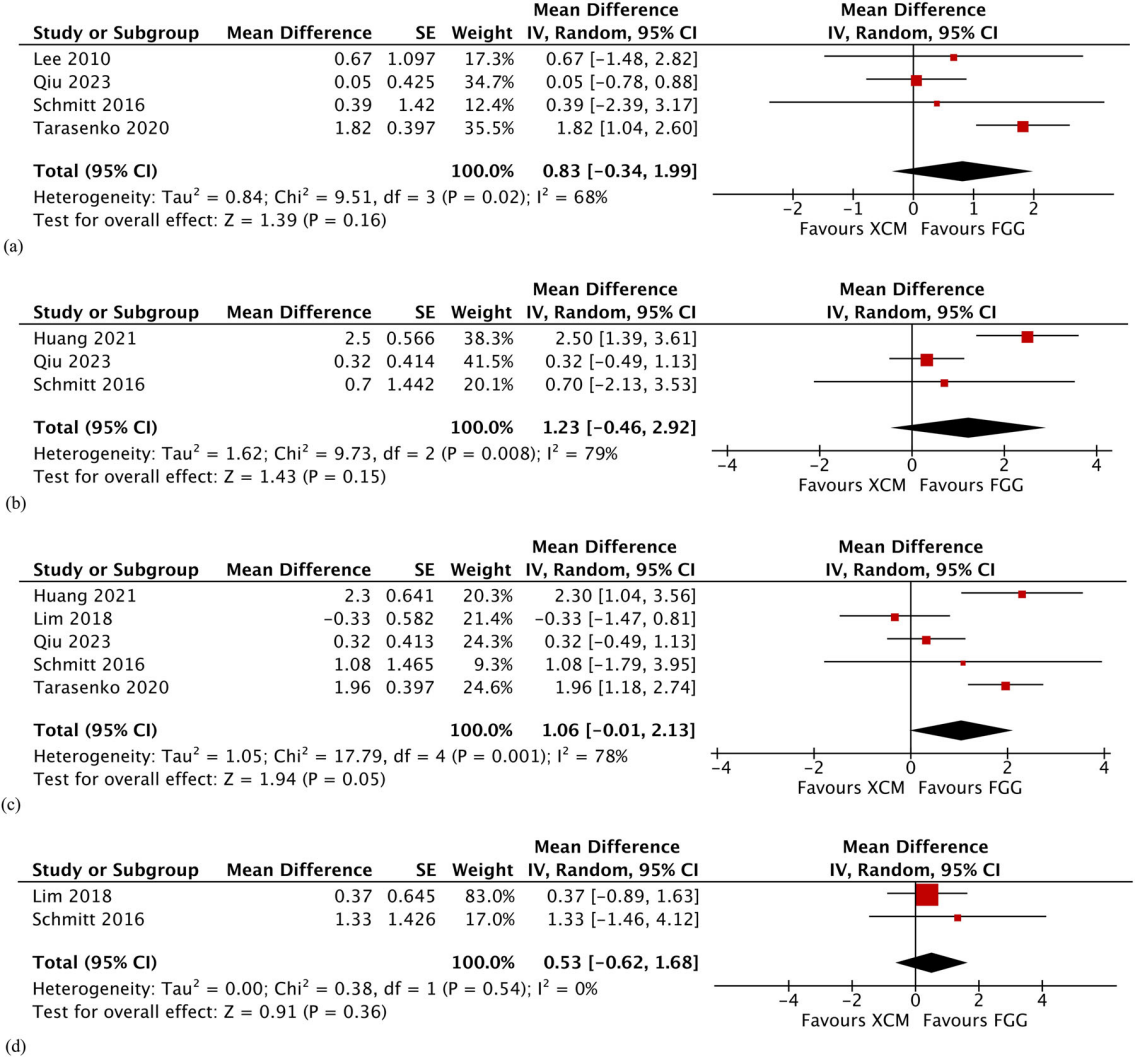
Comparison: Xenogeneic collagen matrix versus free gingival graft. Primary outcome: (a) changes in width of peri‐implant keratinized mucosa at 1 month; (b) changes in width of keratinized mucosa at 2–3 months; (c) changes in width of keratinized mucosa at 6 months; and (d) changes in width of keratinized mucosa at 12 months. τ: Kendall tau; CI: confidence interval; FGG: free gingival graft; IV: inverse variance; SD: standard deviation; XCM: xenogeneic collagen matrix; z: *z*‐test.

All the studies (Huang et al. 2021, Lee, Kim, and Jang 2010, Lim, An, and Lee 2018, Qiu et al. 2023, Schmitt et al. 2016, Tarasenko et al. 2020) reported on changes in width of peri‐implant keratinized mucosa at different time points. At 1 month, sites augmented with FGG were associated with less changes in the gained width of peri‐implant keratinized mucosa compared to those augmented with XCM. The difference, however, was not statistically significant (MD 0.83; 95% CI −0.34 to 1.99; *p* = 0.16; Figure 3a). Significant heterogeneity was detected (*χ* ^2^ = 9.51, *df* = 3 (*p* = 0.02); *I* ^2^ = 63%). Likewise, the difference between the two treatment groups at two to 3 months was not statistically significant (MD 1.23; 95% CI −0.46 to 2.92; *p* = 0.15; Figure 3b), whereas the difference in 6‐month changes was marginally significant, in favor of the FGG group (MD 1.06; 95% CI −0.01 to 2.13; *p* = 0.05; Figure 3c). The 12‐month follow‐up analysis, which included only two studies (Lim, An, and Lee 2018, Schmitt et al. 2016), showed no statistically significant difference between the two groups (MD 0.53; 95% CI −0.62 to 1.68; *p* = 0.36; Figure 3d). Substantial heterogeneity was detected at 2–3 months and the 6‐month meta‐analyses while low heterogeneity was observed at the 12‐month meta‐analysis.

#### 3.9.2 Changes in Thickness of Peri‐Implant Keratinized Mucosa

Two studies (Huang et al. 2021, Qiu et al. 2023) reported on changes in thickness of peri‐implant keratinized mucosa at 6 months. The meta‐analysis showed that the difference between the two groups was statistically significant in favor of FGG (MD 0.51; 95% CI 0.01–1.00; *p* = 0.04; Figure 4a). Substantial heterogeneity was detected (*χ* ^2^ = 3.20, *df* = 1 [*p* = 0.07); *I* ^2^ = 69%).

Figure 4Comparison: Xenogeneic collagen matrix versus free gingival graft. Secondary outcomes: (a) changes in thickness of keratinized mucosa at 6 months; (b) changes in probing pocket depth at 2–3 months; (c) changes in probing pocket depth at 6 months; (d) changes in modified sulcus bleeding index at 1–3 months; (e) changes in modified sulcus bleeding index at 6 months; (f) changes in color; (g) changes in texture; (h) changes in contour; (i) pain score; (j) satisfaction score; and (k) operating time. τ: Kendall tau; CI: confidence interval; FGG: free gingival graft; IV: inverse variance; SD: standard deviation; XCM: xenogeneic collagen matrix; z: *z*‐test.

**Figure:**
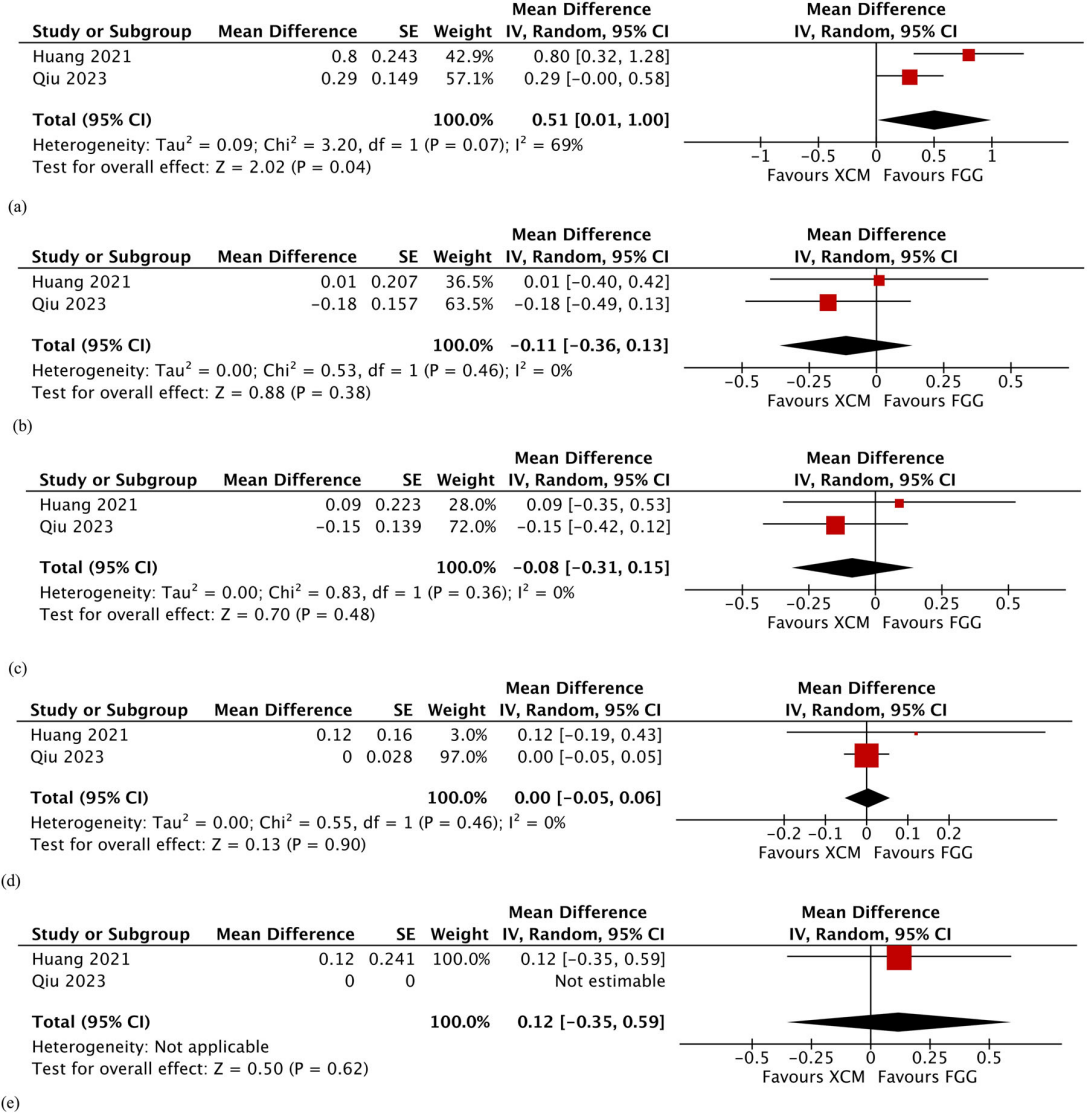

**Figure:**
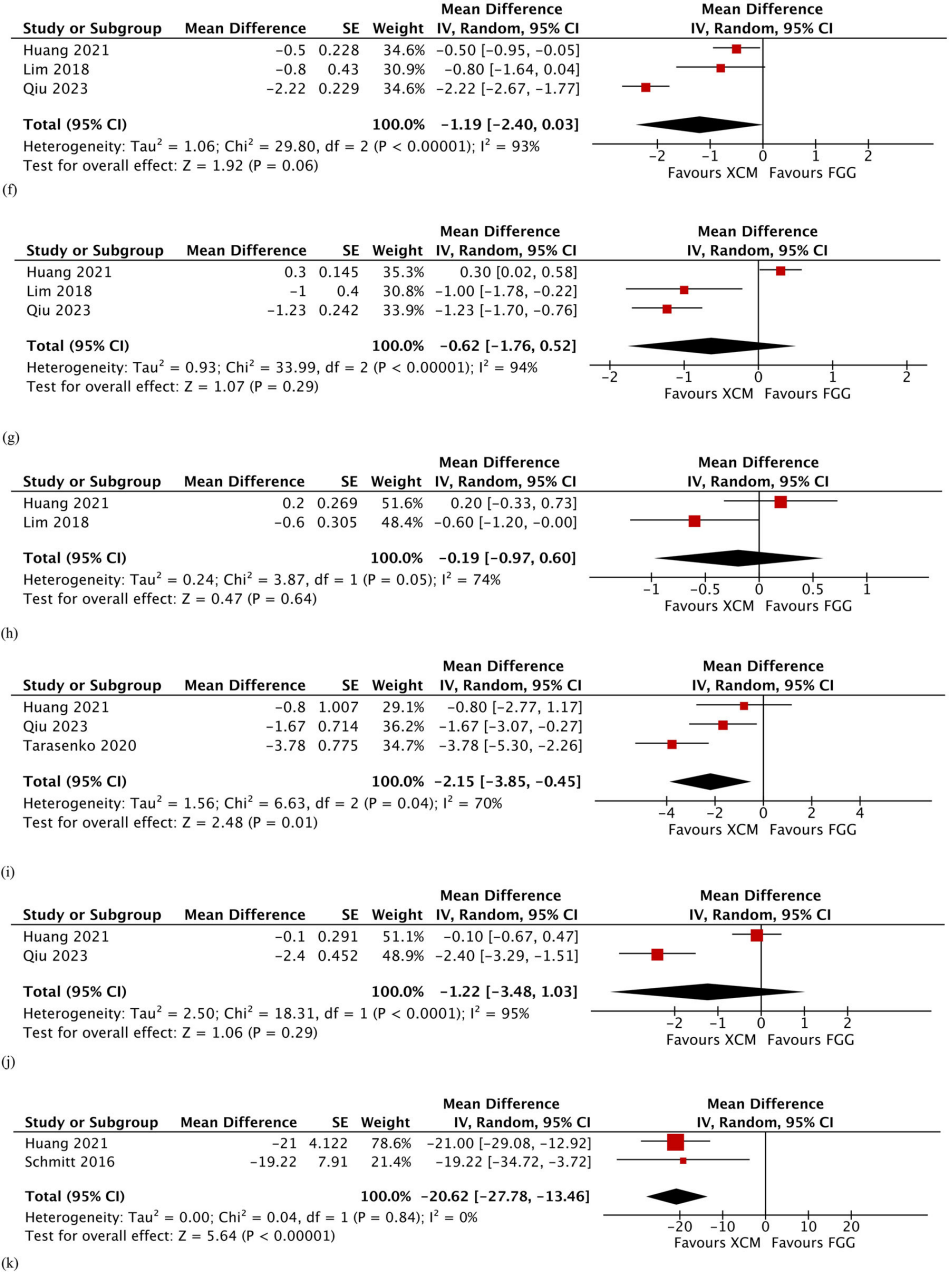

#### 3.9.3 Changes in Periodontal Parameters

Two studies (Huang et al. 2021, Qiu et al. 2023) reported on changes in probing pocket depths and modified sulcus bleeding index. The meta‐analysis showed no statistically significant differences between the two treatment groups in terms of changes in probing pocket depths at 2–3 months (MD −0.11; 95% CI −0.36 to 0.13; *p* = 0.38; Figure 4b) or 6 months (MD −0.08; 95% CI −0.31 to 0.15; *p* = 0.48; Figure 4c). Heterogeneity was not detected at 2–3 months (*χ* ^2^ = 0.53, *df* = 1 [*p* = 0.46]; *I* ^2^ = 0%), while low heterogeneity was observed at the 6‐month meta‐analysis (*χ* ^2^ = 0.83, *df* = 1 [*p* = 0.36]; *I* ^2^ = 0%). Implant sites augmented with XCM and FGG had comparable changes in modified sulcus bleeding index at 1–3 months (MD 0.00; 95% CI −0.05 to 0.06; *p* = 0.90; Figure 4d) and 6 months (MD 0.12; 95% CI −0.35 to 0.59; *p* = 0.62; Figure 4e). Only one study (Qiu et al. 2023) reported on changes in modified plaque index at 1–3 and 6 months with no statistically significant difference between the two treatment groups.

#### 3.9.4 Aesthetic Outcomes

The changes in color and texture were reported in three studies (Huang et al. 2021, Lim, An, and Lee 2018, Qiu et al. 2023), while the changes in contour were reported in two studies (Huang et al. 2021, Lim, An, and Lee 2018). The meta‐analysis showed that implant sites augmented with XCM had more favorable changes in color when compared to implant sites augmented with FGG, but the difference was only marginally significant (MD −1.19; 95% CI −2.40 to 0.03; *p* = 0.06; Figure 4f). Likewise, the changes in texture (MD −0.62; 95% CI −1.76 to 0.52; *p* = 0.29; Figure 4g) and contour (MD −0.19; 95% CI −0.97 to 0.60; *p* = 0.64; Figure 4h) were in favor of XCM treatment group. However, the difference between the two treatment groups was not statistically significant. Substantial heterogeneity was noticed in the meta‐analyses of the three aesthetic outcomes.

#### 3.9.5 Patient‐Reported Outcome Measures

Scores of postoperative pain and satisfaction were reported in three studies (Huang et al. 2021, Qiu et al. 2023, Tarasenko et al. 2020). The XCM treatment group had a significantly lower pain score compared with the FGG treatment group (MD −2.15; 95% CI −3.85 to −0.45; *p* = 0.01; Figure 4i). Substantial heterogeneity was detected (*χ* ^2^ = 6.63, *df* = 2 [*p* = 0.04]; *I* ^2^ = 70%). In terms of satisfaction, there was no significant difference between the two treatment groups (MD −1.22; 95% CI −3.48 to 1.03; *p* = 0.29; Figure 4j).

#### 3.9.6 Operating Time

Two studies (Huang et al. 2021, Schmitt et al. 2016) measured the operating times in minutes. The operating time was significantly shorter in the XCM group compared to the FGG group (MD −20.62; 95% CI −27.78 to −13.46; *p* < 0.0001; Figure 4k). No heterogeneity was detected (*χ* ^2^ = 0.04, *df* = 1 [*p* = 0.84]; *I* ^2^ = 0%).

#### 3.9.7 Sensitivity Analyses

The leave‐one study‐out sensitivity analysis showed that the exclusion of one study (Lim, An, and Lee 2018), judged to be at high risk of bias, has influenced the overall effect size estimate for changes in width of peri‐implant keratinized mucosa at 6 months. The exclusion of this study showed that the difference between the two treatment groups was statistically significant in favor of FGG (Table 5). The meta‐analysis of only randomized controlled trials, rated at low risk of bias, showed that sites augmented with FGG were associated with significantly less changes in the gained width of peri‐implant keratinized mucosa compared to those augmented with XCM at 6 months (MD 1.48; 95% CI 0.24–2.72; *p* = 0.02).

**Table 5:** Leave‐one study‐out sensitivity analysis: Changes in width of peri‐implant keratinized mucosa at 6 months.

**Removed study**	**Overall MD (95% CI)**	***p* value**	**Heterogeneity**
Huang et al. (2021)	0.74 (−0.45, 1.93)	*p* = 0.22	*p* = 0.004; *I* ^2^ = 78%
Lim, An, and Lee (2018)	1.43 (0.34, 2.52)	*p* = 0.01	*p* = 0.01; *I* ^2^ = 72%
Qiu et al. (2023)	1.29 (−0.03, 2.61)	*p* = 0.06	*p* = 0.005; *I* ^2^ = 77%
Schmitt et al. (2016)	1.06 (−0.12, 2.23)	*p* = 0.08	*p* = 0.0005; *I* ^2^ = 83%
Tarasenko et al. (2020)	0.76 (−0.43, 1.95)	*p* = 0.21	*p* = 0.02; *I* ^2^ = 70%

Abbreviations: CI: confidence interval; MD: mean difference.

## 4 Discussion

### 4.1 Summary of Main Results

The present systematic review compared implant sites augmented with FGG to those augmented with XCM, before commencing prosthetic implant treatment, in terms of changes in width of the keratinized mucosa, the thickness of keratinized mucosa, probing pocket depths, modified bleeding and plaque indices, aesthetic outcomes, patient‐reported outcome measures, and operating time. Sites augmented with FGG showed less contraction compared with XCM, but the difference between the two groups reached a marginal statistical significance at 6 months. The difference between the two groups in terms of changes in thickness of keratinized mucosa at 6 months was statistically significant in favor of FGG. On the other hand, the use of XCM to augment keratinized mucosa was associated with significantly shorter operating time, significantly lesser postoperative pain and marginally significant improvement in color change. In terms of patient satisfaction and changes in contour and texture of augmented sites, the results were comparable between the two groups.

### 4.2 Quality of Evidence

Three of the included studies in the present systematic review were not randomized and were rated at high risk of bias (Lee, Kim, and Jang 2010, Lim, An, and Lee 2018, Schmitt et al. 2016). The remaining three studies (Huang et al. 2021, Qiu et al. 2023, Tarasenko et al. 2020), however, were randomized and were judged at low risk of bias. A sensitivity analysis of the primary outcome was performed, and the removal of either one study (Lim, An, and Lee 2018) or the inclusion of only randomized controlled trials had an impact on the changes in width of keratinized mucosa at 6 months in favor of FGG. The overall certainty of evidence for the primary outcome looking at changes in width of keratinized mucosa at 1 month was judged moderate, whereas the evidence quality for the same outcome at 2–12 months was judged low. The quality of evidence for the remaining outcomes, including aesthetic outcomes and patient‐reported outcome measures, varied from moderate to low due to substantial statistical heterogeneity, lack of blinding, and imprecision. Despite the standardization in selection criteria of participants, surgical techniques, and timings of soft tissue augmentation, the inclusion of both anterior and posterior implant sites and methods of assessment (periodontal probe vs. cone beam computed tomography) could be regarded as potential sources of the observed heterogeneity.

### 4.3 Applicability of Evidence

The present systematic review has shown that both FGG and XCM are effective techniques for augmenting the keratinized mucosa in implant sites with shallow vestibules and insufficient keratinized mucosa. Our findings showed that the gain in width and thickness of peri‐implant keratinized mucosa was in accordance with other studies (Sanz et al. 2009, Schmitt et al. 2013, Solonko et al. 2022). XCM showed more shrinkage in width and thickness than FGG at different time points. Although the difference was not statistically significant at 1–3 or 12 months, it did reach a marginal statistical significance at 6 months. Moreover, the changes in thickness at 6 months were significantly more in the XCM group. The shrinkage of XCM has previously been reported in other studies (Nevins et al. 2011, Sanz et al. 2009), indicating that there is low to moderate evidence to suggest that FGG is more likely to maintain its volume up to 12 months as compared to XCM. It has been suggested that the shrinkage of XCM can be minimized by using a strip of FGG at the apical portion of the recipient bed that served as a mechanical barrier and cell source, maintaining the apical displacement of the desired mucogingival junction position, thereby generating the desired keratinized mucosa (Urban et al. 2015, 2019); however, the proposed procedure was outside the scope of the present review to assess.

Another vital aspect that contributes to maintenance of peri‐implant health and correction of aesthetic outcomes around implants is the thickness of the peri‐implant keratinized mucosa. Although there is no consensus on the required thickness of peri‐implant keratinized mucosa for optimal aesthetic and functional outcomes, a thick phenotype of ≥ 2 mm has been associated with lower incidence of mucosal recession and marginal bone loss (Tavelli et al. 2021, Thoma et al. 2018). Additionally, a thicker peri‐implant soft tissue also aids in masking the “gray zone” of the implant–abutment interface. This meta‐analysis suggests that XCM might not be an alternative to FGG, as FGG ensured greater stability of width and thickness of keratinized mucosa over 12‐month observation time, aligning with prior findings on soft tissue augmentation around dental implants (Bassetti et al. 2016). Alternative procedures to increase the keratinized mucosal thickness around dental implants such as the bilaminar placement of subepithelial connective tissue graft or other substitutes with overlying pedicled graft have previously been described (Thoma et al. 2018). Their effectiveness, however, is outside the scope of this review.

XCM could be the preferred option in terms of patient perception of pain as pain score was significantly less as compared with the FGG group where a donor site is required (i.e., palate). This is in agreement with several studies (Schmitt et al. 2013, Solonko et al. 2022, Tonetti et al. 2018) in which the presence of a second surgical site increased postoperative pain and discomfort. As previously reported (Tavelli et al. 2021), the present review confirmed that the augmentation of peri‐implant keratinized mucosa did not have any adverse events on peri‐implant health as assessed by probing pocket depths, bleeding, or plaque indices. The aesthetic outcomes were in favor of XCM, as XCM achieved a better match in color, texture, and contour when compared to FGG. The difference was only marginally significant in terms of color, but the overall aesthetic appearance of XCM grafted sites corroborated with other findings (Nevins et al. 2011, Sanz et al. 2009, Schmitt et al. 2013, Urban et al. 2015), which showed that XCM acts as a scaffold for oral keratinocytes that differentiate into matched keratinized mucosa. In contrast, FGG maintained its distinct color and texture, which is often described as a “tire‐patch” appearance (Yukna et al. 1977).

### 4.4 Agreements and Disagreements With Other Reviews

Despite the abundance of reviews on soft tissue augmentation around dental implants (Atieh and Alsabeeha 2020, Tavelli et al. 2021, Thoma et al. 2018, Zucchelli et al. 2020), only two (Bassetti et al. 2016, Lin et al. 2018) evaluated the impact of soft tissue augmentation at the time of uncovering dental implants or before the placement of the final prosthesis. The first review (Bassetti et al. 2016) was a systematic review that evaluated the impact of using different autogenous tissue grafts and substitutes on the dimensional changes of keratinized mucosa, aesthetics, and periodontal parameters. Meta‐analysis was not attempted due to heterogeneity of the surgical techniques and augmentation materials used. The authors concluded that autogenous tissue grafts were still the gold standard in terms of long‐term tissue stability. The second review of Lin et al. (2018) assessed the effect of timing of soft tissue augmentation on stability of width and thickness of keratinized mucosa. That review conducted meta‐analyses on four studies that described augmentation of keratinized mucosa after implant placement and before placement of the final prosthesis. Two of the included four studies were from the same trial that compared FGG to XCM. The authors concluded that there were no differences between simultaneous or staged soft tissue augmentation in terms of changes in soft tissue width and thickness. Neither of the reviews made reference to patient‐reported outcome measures and did not provide any specific conclusions regarding the comparative effects of FGG versus XCM.

The present systematic review has several limitations that are mainly related to the retrospective and non‐randomized design of some included studies and insufficient data on all the outcomes set for the review. Nevertheless, the meta‐analysis, particularly when non‐randomized controlled trials were excluded, confirmed the beneficial effects of FGG, as compared to XCM, in preserving the gain in width and thickness of keratinized mucosa. That positive impact, however, was on the expense of the aesthetics and perception of postoperative pain. It needs to be recognized that the existing literature on the influence of soft tissue augmentation before the placement of the final prosthesis is currently limited and additional long‐term studies are still needed.

## 5 Conclusions

Within the limitation of this review, the augmentation of keratinized mucosa using FGG before the placement of the final prosthesis may have short‐term positive effects on soft tissue thickness. XCM might be considered in aesthetically demanding implant sites and where patient comfort or shorter surgical time is a priority. The evidence support, however, is of low to moderate certainty, and therefore, further studies are needed to support the findings of the present review.

## Author Contributions

Momen A. Atieh contributed to the concept/design, data collection, data analysis/interpretation, drafting of the article, critical revision of the article, and approval of the article. Maanas Shah was involved in data analysis/interpretation, critical revision of the article, and approval of the article. Suhailah Alshaali, Abeer Hakam, Reem Kasouha, and Andrew Tawse‐Smith critically revised the article and approved the article. Nabeel H.M. Alsabeeha was involved in data collection, data analysis/interpretation, critical revision of the article, and approval of the article.

## Conflicts of Interest

The authors declare no conflicts of interest.

## Data Availability

The data that support the findings of this study are available on request from the corresponding author. The data are not publicly available due to privacy or ethical approval.
